# Requirement of histone deacetylase activity for the expression of critical photoreceptor genes

**DOI:** 10.1186/1471-213X-7-78

**Published:** 2007-06-29

**Authors:** Bo Chen, Constance L Cepko

**Affiliations:** 1 Department of Genetics and Howard Hughes Medical Institute, Harvard Medical School, 77 Avenue Louis Pasteur, Boston, MA 02115, USA

## Abstract

**Background:**

Histone deacetylases (HDACs) play a major role in the regulation of gene transcription, often leading to transcriptional repression, as well as other effects following deacetylation of non-histone proteins.

**Results:**

To investigate the role of HDACs in the developing mammalian retina, a general inhibitor of HDACs, trichostatin-A (TSA), was used to treat newborn murine retinae in explant cultures. Inhibition of HDAC activity resulted in a reduction in RNA levels for genes that regulate retinal development, as well as cell cycle regulators. Several of the genes encode transcription factors essential for rod photoreceptor development, Otx2, Nrl, and Crx. Using luciferase reporter assays, the promoter activity of both Nrl and Crx was found to be compromised by HDAC inhibition. Furthermore, downregulation of gene expression by HDAC inhibition didn't require de novo protein synthesis, and was associated with hyperacetylation of histones and non-histone proteins. Finally, HDAC inhibition in retinal explant cultures resulted in increased cell death, reduction in proliferation, a complete loss of rod photoreceptors and Müller glial cells, and an increase in bipolar cells.

**Conclusion:**

HDAC activity is required for the expression of critical pro-rod transcription factors and the development of rod photoreceptor cells.

## Background

Histone acetylation is a posttranslational modification that leads to changes in chromatin structure and transcription. The acetylation level of histones is governed by the opposing effects of two enzymes, histone acetyltransferases (HATs) and histone deacetylases (HDACs), which are responsible for adding and removing acetyl groups from lysine residues, respectively. It is generally believed that HDACs lead to transcription repression as histone hypoacetylation results in a tightly packaged chromatin structure, denying accessibility to transcription regulatory proteins. Histone hyperacetylation relaxes chromatin structure and is associated with increased transcriptional acitivity [1-4]. However, histones are not the sole target of HDACs. Other non-histone HDAC substrates include transcription factors, such as E2F1, MyoD, GATA-1, and p53 [5-9], as well as proteins in the cytoplasm, such as tubulin and hsp90 [10-14].

Mammalian HDACs can be classified into two groups based upon their structure and sequence homology to their yeast counterparts. Class I HDACs (HDAC 1, 2, 3, 8) contain a single catalytic domain and are ubiquitously expressed in all tissues. The subcellular localization of Class I HDACs is almost exclusively in the nucleus. Class II HDACs (HDAC 4, 5, 6, 7, 9, 10) consist of a C-terminal catalytic domain and an N-terminal portion that is used to mediate interactions with other proteins. Class II HDACs are preferentially expressed in cardiac muscle, skeletal muscle, and brain. Interaction of Class II HDACs with MEF2 silences the expression of MEF2 target genes, thus suppressing myocyte differentiation [15-17]. Phosphorylation of Class II HDACs by CaMK and other kinases causes their shuttling out of the nucleus and accumulation in the cytoplasm, thus releasing their suppression of MEF2 target genes [18,19]. Although substantial evidence is available that HDACs play a role in transcription repression, recent findings clearly demonstrate that HDACs can act as transcription activators as well. SRC promoter repression by HDAC inhibition is one example [20]; blockade of cytokine-inducible gene expression and antiviral immune response by the loss of HDAC activity is another [21-24].

The retina is a highly-organized tissue specialized for sensing light and processing the signal that originates from activated photoreceptors. The mature retina is composed of 6 neuronal and 1 glial cell type. Each of the different cell types is generated in a specific time window from multipotent retinal progenitor cells. The cell fate decision made by a retinal cell depends upon both the intrinsic properties of its progenitor as well as environmental cues [25,26]. We have proposed that progenitor cells go through a progression of competency states, each defined by the ability to make different retinal cell types [25]. Each competency state is likely controlled by a distinct network of transcription factors. For example, a specific set of transcription factors may allow a multipotent progenitor cell to respond to a particular extrinsic cue to produce a rod photoreceptor. Rod photoreceptor cells are the most abundant cell type in the rodent retina; they are almost continually produced from retinal progenitor cells during the embryonic and neonatal period, overlapping the production of almost all the other cell types. A terminally differentiated rod photoreceptor cell can be identified by the expression of the visual pigment protein, Rhodopsin. We and others have identified Otx2, Nrl, and Crx, as critical transcription factors required for rod photoreceptor development [27-32]. Moreover, Nrl and Crx physically interact with each other to activate the Rhodopsin promoter [33]. However, the mechanisms controlling the expression of these transcription factors remain largely unknown.

To probe a potential role of HDACs in the regulation of retinal gene expression, we applied TSA, a potent drug that inhibits all HDACs, to retinal explant cultures and assayed gene expression changes. HDAC inhibition led to a marked reduction in gene expression for all three (Otx2, Nrl, Crx) transcription factors required for rod photoreceptor development. By microarray analysis, the effect of HDAC inhibition on gene expression was observed to be mainly restricted to a specific subset of genes essential for retinal development, rather than a global alteration in a large number of genes. These data suggest a net positive effect of HDACs on retinal gene expression. Downregulation of gene expression by TSA took place within 3 hours, and the TSA effect didn't require new protein synthesis. Furthermore, a promoter-reporter analysis showed that HDAC activity is a positive regulator of the promoter activity of Nrl and Crx. Interestingly, HDAC inhibition in retinal explant cultures blocked rod photoreceptor and Müller glial development, while increasing the number of bipolar cells, suggesting a role for HDACs in cell fate decisions.

## Results

### HDAC activity is required for the expression of critical genes for rod photoreceptor development

To determine if HDACs might play a role in retinal development, RT-PCR was used to examine the expression of members of Class I (HDAC1 and 3) and Class II HDACs (HDAC4, 5, and 6) in the developing mouse retina. RNA samples were analyzed at postnatal day 2 (P2), when rod photoreceptor, bipolar and Müller glial cells are produced [34]. Full-length coding regions of HDAC1 and HDAC3, as well as ~200 bp from the 3' regions of HDAC4, 5, and 6, were amplified. The identity of amplified cDNAs was confirmed by DNA sequencing. The results indicate that HDAC1 and 3 (Class I) and HDAC4, 5, and 6 (Class II HDACs) are expressed in the developing murine retina (data not shown).

**Figure 1:**
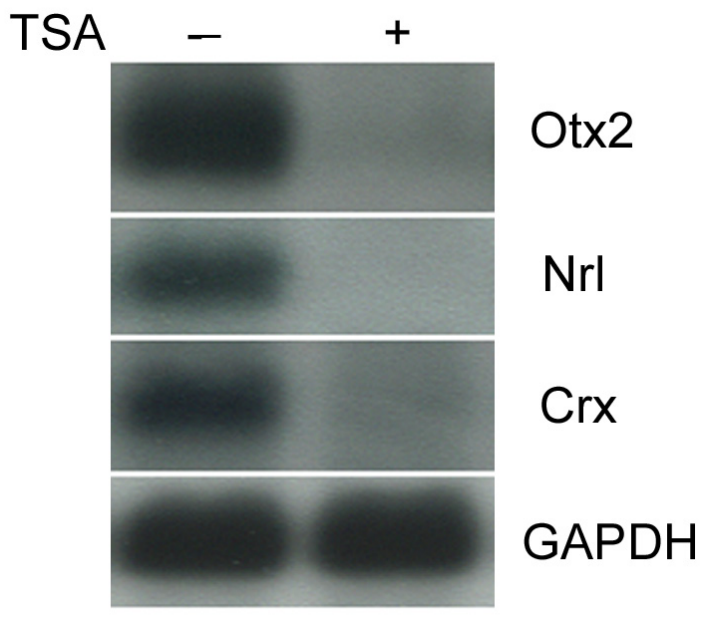
Northern blot analysis of the expression of Otx2, Nrl, and Crx when P2 mouse retinal explants were treated with 1 μM TSA or DMSO for 3 hours.

Rod photoreceptors constitute the major cell type in the murine retina [34]. Proper differentiation of rods requires the action of the transcription factors, Otx2, Nrl, and Crx. Compromise of any of these factors results in a total loss of, or severely compromised, rod photoreceptor development. To probe the role of HDACs in the development of rod photoreceptors, TSA, a potent inhibitor of both Class I and Class II HDACs, was used to treat P2 mouse retinal explant cultures. After 3 hours of TSA treatment, a significant reduction was seen in the levels of RNA for Otx2, Nrl, and Crx, while the expression of a house keeping gene, GAPDH, remained unchanged (Fig. 1). A similar level of downregulation was observed for these three genes using two other HDAC inhibitors, valproic acid (VPA) and sodium bytyrate (N_a_B), for a 3-hour treatment in P2 mouse explant cultures (data not shown).

### Inhibition of HDACs causes downregulation of a specific subset of genes

**Figure 2:**
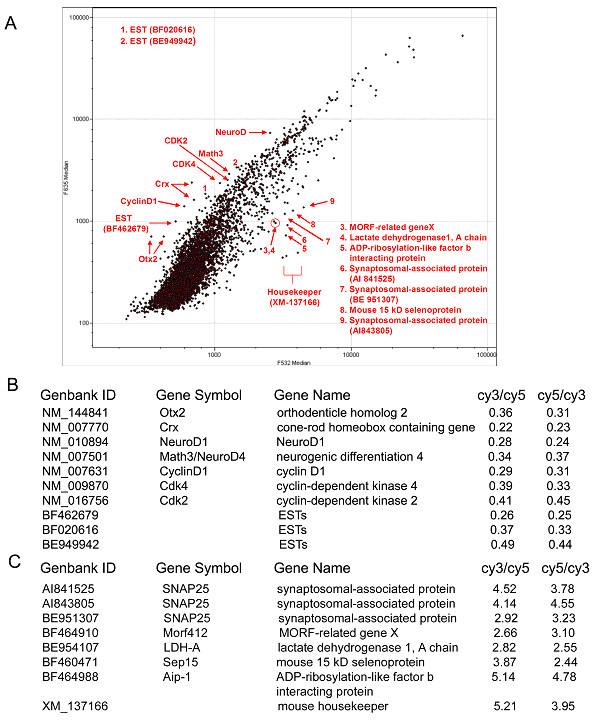
Effects of HDAC inhibition on retinal gene expression. P2 mouse explant cultures were treated with 1 μM TSA or DMSO vehicle control for 3 hours. Total RNA was extracted and labeled with cy3 or cy5 for TSA and DMSO treatments, respectively. cDNA microarray analysis is shown by a scatter plot in (A). TSA-downregulated genes are distributed above the 45° diagonal; and TSA-upregulated genes are distributed below it. TSA-downregulated genes listed in (B) encompass photoreceptor genes (Otx2 and Crx) and proneural genes (NeuroD1 and Math3/NeuroD4) as well as cell cycle regulators (CyclinD1, Cdk4, and Cdk2). TSA-upregulated genes shown in (C) include a synaptosomal-associated protein, SNAP25. Values given (B and C) represent the fold change upon TSA treatment. The second value (cy5/cy3) shows the fold change in a dye swap experiment.

To investigate whether the inhibition of HDACs caused a global change in gene expression, or whether the effect was restricted to a subset of genes, a cDNA microarray assay was performed comparing expression profiles of TSA treated and vehicle DMSO treated samples. Three hours after the drug treatment, total RNA was extracted from P2 mouse retinae. TSA treated samples were labeled by cy3, DMSO treated samples were labeled by cy5. Labeled cDNAs were hybridized to a cDNA chip (Experimental Procedures) and plotted with a log ratio (Fig. 2A). A dye swap experiment also was performed in which each probe was labeled with the other dye. Widespread gene expression changes were not observed; changes were restricted to a subset of genes. Among those downregulated (points distributed above the 45° degree diagonal) by TSA, were Otx2 and Crx, as originally identified by the candidate approach shown in Fig. 1. TSA downregulated genes (Fig. 2B) can be divided into two groups. Group 1 consists of retinal development regulators, including Otx2, Crx, Neurod1, and Neurod4/Math3. Neurod1 and Neuod4/Math3 are two other transcription factors that positively regulate neuron development, including rod photoreceptor development [35-37]. Group 2 consists of cell cycle regulators, including CyclinD1, Cdk4, and Cdk2. The downregulation of cell cycle regulators is consistent with the known anti-proliferation effect observed following inhibition of HDAC activity [38,39]. Recent studies showed that HDAC1 mutants manifested defects in the retinal cell proliferation and cell-cycle exit in zebrafish([40,41]. Among TSA-upregulated genes (points distributed below the 45° diagonal), was a gene encoding a synaptosomal-associated protein, SNAP25 (Fig. 2C), suggesting HDACs may also play a role in synaptogenesis. Through the microarray analysis, it seems that HDAC activity is required for regulation of a subset of genes expressed during retinal development.

### HDAC activity is required for maintaining the expression of pro-rod genes

**Figure 3:**
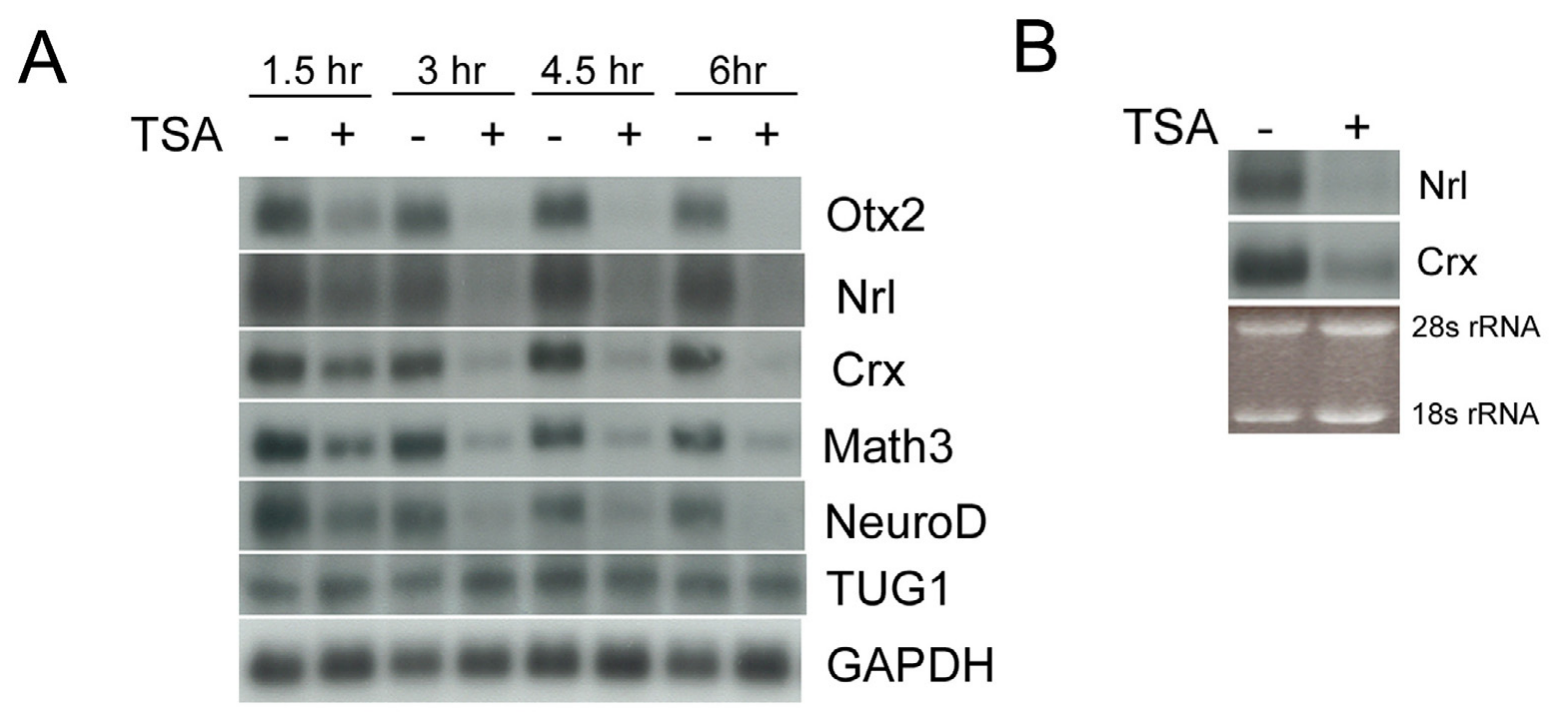
(A) Northern blot analysis of the time course of changes in retinal gene expression when HDAC activity was inhibited in P2 mouse retinal explants. (B) Effect of HDAC inhibition on Nrl and Crx expression in P21 mouse retinal explants by Northern blot analysis.

The group of genes specifically downregulated by HDAC inhibition that have known functions for retinal development were studied further. To validate the microarray results, P2 mouse retinal explants were cultured in the presence or absence of TSA, followed by Northern blot analysis to monitor the time course of change in gene expression for Otx2, Nrl, Crx, Neurod1 and Neurod4/Math3. Downregulation of gene expression began to be seen within 1.5 hours after TSA treatment. The effect was obvious after 3 hours, and maintained at later time points (Fig. 3A). The expression of TUG1, a putative non-coding RNA involved in photoreceptor differentiation [42], was not affected by TSA, nor was the expression of GAPDH (Fig. 3A). Since the TSA effect was rather robust for the developing retina, it was of interest to determine whether HDAC activity is required for the maintained expression of these genes in a mature retina. Nrl and Crx expression levels in P21 mouse retinae, when retinal differentiation is complete, were analyzed. Nrl and Crx were selected for further analysis not only because they promote rod photoreceptor formation, but they act together to transactivate the promoter of the rhodopsin gene [33], a functional marker for rod photoreceptor cells. P21 mouse retinal explants were cultured in the presence of or absence of TSA for 24 hours. Using Northern blot analysis, it was found that the expression of Nrl was reduced to a minimum level, and only a trace amount of Crx mRNA remained (Fig. 3B), following TSA treatment.

### HDACs positively regulate the promoter activity of Nrl and Crx

**Figure 4:**
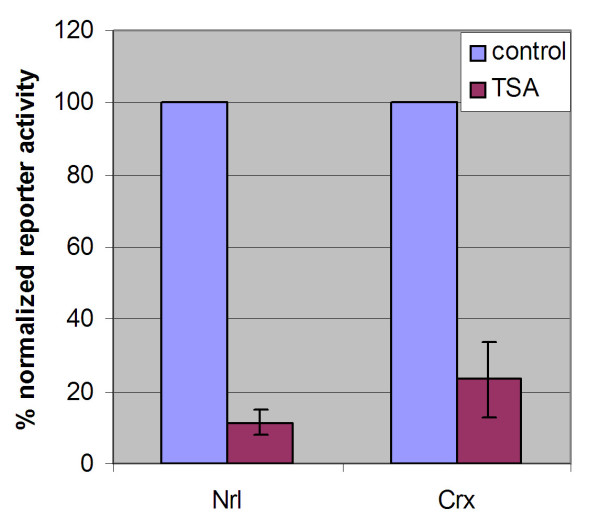
Luciferase reporter analysis of Nrl and Crx promoters in P2 mouse retinal explants (n = 3). Firefly luciferase reporter constructs driven by Nrl or Crx promoter were electroporated into retinal explants. Co-electroporation of a renilla luciferase reporter construct driven by a ubiquitous CAG promoter was used to normalize differences in the transfection efficiency. TSA-treated reporter activity is expressed as a percentage of the normalized DMSO-treated reporter activity.

To investigate the mechanism through which the expression of rhodopsin activators is downregulated by HDAC inhibition, the promoter activity of Nrl and Crx was examined upon HDAC inhibition using a luciferase reporter assay. A 7 kb and 5 kb upstream regulatory sequence was isolated for Nrl and Crx, respectively. Nrl and Crx sequences were cloned into the pGL3-basic firefly luciferase vector (Promega). Luciferase reporters were transfected into P2 mouse retinal explants by in vitro electroporation. A pCAG-renilla luciferase expression vector was co-electroporated to allow for normalization of the transfection efficiency. Electroporated retinae were allowed to recover overnight in culture. TSA was added for a 24 hour treatment before retinal lysates were subjected to a dual luciferase assay. Shown in Fig. 4, the promoter activity of Nrl in TSA-treated retinae was reduced to 11.5 ± 3.3% of the normalized control with DMSO treatment (n = 3). The promoter activity of Crx in TSA-treated retinae was reduced to 23.5 ± 10.5% of the normalized control with DMSO treatment (n = 3).

### The effect of HDAC inhibiton on gene expression doesn't require de novo protein synthesis and is associated with hyperacetylation of multiple cellular proteins

**Figure 5:**
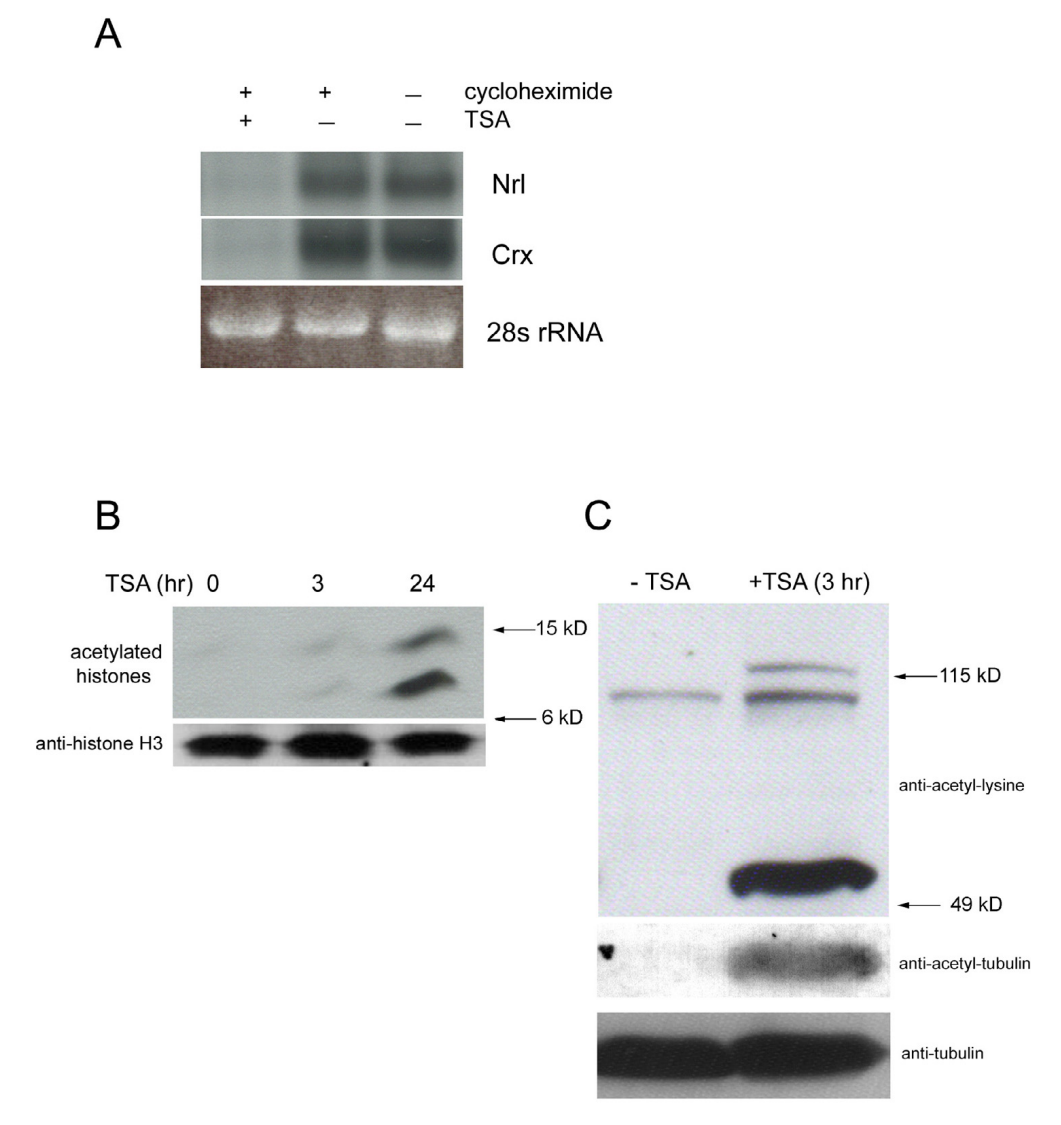
(A) Effect of protein synthesis inhibition on the downregulation of Nrl and Crx by TSA. P2 retinal explant cultures were treated with TSA, TSA plus cycloheximide, or DMSO for 3 hours, followed by Northern blot analysis. (B) Effect of HDAC inhibition on histone acetylation by western blot analysis. (C) Effect of HDAC inhibition on the acetylation of non-histone proteins by western blot analysis. The blot was sequentially probed with a pan acetyl-lysine antibodyn (top blot), an acetyl-tubulin antibody (middle blot), and an α-tubulin antibody (bottom blot).

To further understand HDAC regulation of gene expression, the question of whether the synthesis of new protein(s) was necessary for the inhibition of gene expression by TSA was examined. To address this question, TSA was co-applied to mouse retinal explant cultures with a protein synthesis inhibitor, cycloheximide, for 3 hours. The expression of Nrl and Crx was assayed on Northern blots. Co-treatment with cycloheximide did not change the effect exerted by TSA. The expression of both Nrl and Crx was almost undetectable when TSA and cycloheximide were co-applied, whereas retinae treated with cycloheximide alone showed unaltered expression levels for Nrl and Crx (Fig. 5A), compared to untreated controls. Apparently, de novo protein synthesis was not required for the downregulation of Nrl and Crx expression by HDAC inhibition.

To identify HDAC target proteins which may be mediating the TSA effect, a pan-acetyl-lysine antibody was used to examine acetylated proteins in western blots when P2 mouse retinae were treated by TSA for various time points. As expected, a slightly increased histone acetylation was seen 3 hours after TSA treatment, and a pronounced histone acetylation was seen after 24 hours (Fig. 5B). In contrast to the slow kinetics of histone acetylation, robust acetylation was seen only 3 hours after TSA treatment for multiple non-histone targets, including α-tubulin with the molecular weight about 50 kD, which is a substrate for HDAC6, and two other proteins with molecular weights close to 115 kD (Fig. 5C). The fast hyperacetylation of these non-histone proteins coincided with the quick disappearance of the levels of RNA for some retinal genes by TSA, which became apparent in 3 hours.

### HDAC inhibition affects development of several retinal cell types

Six neuronal and one glial cell type are produced from retinal progenitor cells, with 4 cell types born in the neonatal period in mice: rod photoreceptor cells, bipolar cells, amacrine, and Müller glial cells. Since the expression of critical genes for rod photoreceptor development was dependent on the activity of HDACs, the effects of TSA on rod development was assayed in an intact organ culture system. P2 mouse retinal explants were cultured in the presence or absence of TSA until the equivalent of P10. Retinal explants were then dissociated and subjected to immunostaining with various cell type-specific antibodies.

**Figure 6:**
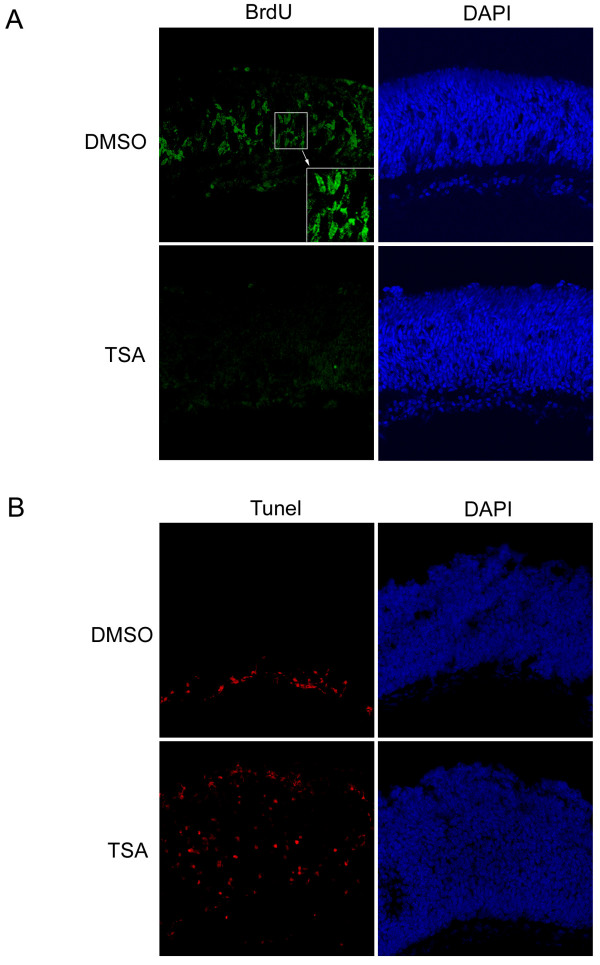
HDAC inhibition leads to cell cycle arrest and apoptotic cell death. P2 mouse retinal explants were cultured in the presence of 100 nM TSA or DMSO for 20 hours before one hour of BrdU labeling, shown in (A), and TUNEL cell death detection, shown in (B).

It was observed that after 8 days in culture, the total number of cells from the TSA treated retinal explants was reduced by 32.2 ± 5.2% (n = 3) compared to the DMSO-treated controls, assessed by counting the number of dissociated cells. The reduced cell number may reflect the effect of HDAC inhibition on cell proliferation and/or cell death. To distinguish between these possibilities, P2 mouse retinal explants were cultured in the presence or absence of TSA for 20 hours before cell cycle progression and cell death were assayed by BrdU labeling of mitotic cells and TUNEL labeling of apoptotic cells, respectively. Consistent with the finding that TSA treatment downregulates cell cycle regulators in the microarray analysis (Fig. 2), HDAC inhibition was found to cause a complete cell cycle arrest; no BrdU positive cell was detected in the TSA treated retinal explants compared to the controls (Fig. 6A). At the same time, while the DMSO-treated retinal explants displayed minimal cell death in the ganglion cell layer, many more apoptotic cells were found to be distributed across the retina (Fig. 6B) when HDAC activity was inhibited by TSA. Therefore, the decreased total cell number by TSA treatment was accounted for by the combined effects of HDAC inhibition that led to both cell cycle arrest and increased cell apoptosis. However, the remaining TSA-treated cells looked morphologically normal when they were dissociated after 8 days in culture.

**Figure 7:**
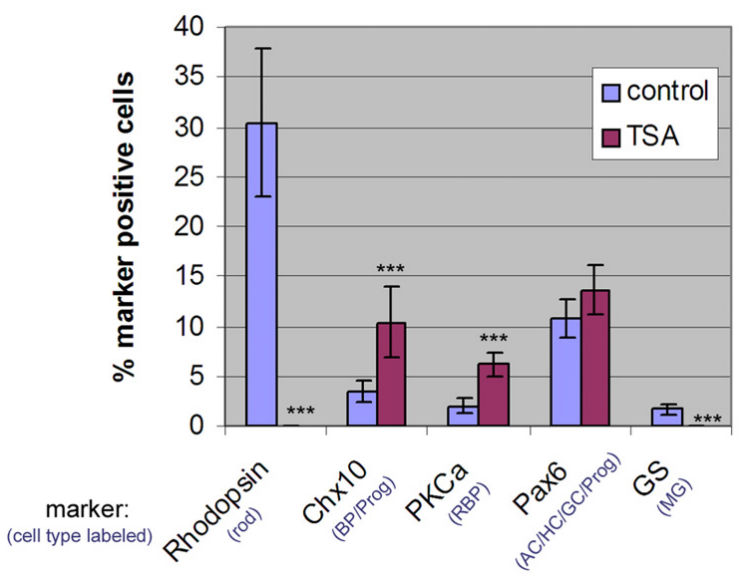
Effect of HDAC inhibition on the development of retinal cell types. P2 mouse retinal explants were cultured in the presence of 100 nM TSA or DMSO. After 8 days in culture, cells were dissociated and immunostained with cell type-specific antibodies. Marker positive cells were counted for each antibody from 3 independent experiments. Eight hundred to 1000 cells were counted for each antibody in each experiment. In all of the three experiments, not a single cell was detected as Rhodopsin or GS positive when retinal explants were treated with TSA. Significance analysis was performed using a Student's t-Test. *** p < 0.05. (BP-bipolar cells, Prog-progenitor cells, RBP-rod bipolar cells, AC-amacrine cells, HC-horizontal cells, GC-ganglion cells, MG- Müller glial cells.)

To assay the effects of TSA on retinal cell types, cultured cells were dissociated and stained with several cell type-specific antibodies. The immunostaining results were quantified (Fig. 7) by counting marker positive cells from 3 independent experiments, with 800–1000 cells counted for each marker in each experiment. For DMSO treated retinal explants, ≈ 30.4 ± 7.4% cells were clearly Rhodopsin positive at P10. However, not even a single Rhodopsin positive cell was detected in TSA treated cultures. To detect bipolar cells, an antiserum to Chx10, which is expressed in bipolar and progenitor cells, was used. The percentage of Chx10 positive cells in TSA treated samples was increased by about 3 fold to 10.4 ± 3.5% from 3.5 ± 1.1% in controls. To check if different types of bipolar cells were increased, dissociated cells were also stained for PKCα, a marker of rod bipolar cells. In line with an increase in Chx10 staining, there was a significant increase in the percentage of PKCα positive cells when retinal explants were cultured in the presence of TSA, 6.2 ± 1.2% compared to 2.0 ± 0.8% in controls. The inhibition of HDACs was also found to suppress the expression of glutamine synthetase, a marker for Müller glial cells. Inhibition of Müller glial development by TSA was confirmed with antisera to two other Müller genes, CyclinD3 and ApoE (data not shown). To check for changes in progenitor cells, and/or cell types born prenatally, antisera to Pax6, which labels progenitor cells, amacrine cells, ganglion cells, and horizontal cells, was used. There was no significant difference in the number of Pax-6 expressing cells in the TSA-treated retinas (13.6 ± 2.5%) versus controls (10.76 ± 2.0%).

## Discussion

Retinal progenitor cells are multipotent throughout development, producing cell types in a conserved order. Early progenitor cells give rise to early born cell types: the ganglion cells, horizontal cells, cones and amacrine cells. Late progenitor cells produce late born cell types: the bipolar cells and Müller glial cells. Rod photoreceptors, the major cell type in the murine retina, are generated both before and after birth, with peak production taking place around birth [43]. This process is likely controlled by a network of transcription factors that must be expressed at a particular time and in specific cells. For instance, rod photoreceptor development requires activation of Otx2, followed by the expression of Nrl and Crx. Loss of Otx2 or Nrl leads to loss of rod photoreceptor cells, while loss of Crx leads to a failure in differentiation of rod and cone photoreceptor cells [27,28,32]. Neurod1 also promotes rod photoreceptor development, as overexpression of Neurod1 favors the production of rods at the expense of bipolar cells and Müller glia, and Neurod1 is required for the survival of rod photoreceptor cells [35]. TSA treatment led to a reduction in the expression of all of these key regulators of rod development, and of rod development itself. Downregulation of these regulators took place quickly, within 3 hours, before cell death was observed, 20 hours after TSA treatment. As there was not a general downregulation of gene expression observed on the microarrays, and the effect on the pro rod genes was so rapid, it is unlikely that the effect of TSA on the expression of these key regulators resulted from the cell death effect exerted by TSA. In addition, TSA led to an inhibition of Müller glia development. This may hold generally true in the central nervous system, as inhibition of HDAC activity in multipotent hippocampal neural progenitor cells inhibits glial cell formation [44]. TSA treatment also led to a reduction in total cell counts due to reduced cell proliferation and increased cell apoptosis. However, the observed approximately 30% reduction in cell numbers does not explain a 3 fold increase in bipolar cells when HDAC activity was inhibited by TSA in the retinal explant cultures. Taken together, these results suggest that loss of HDAC activity can drive the bipolar cell fate at the expense of other fates. It is interesting to consider this effect in light of the fact that all proliferation was inhibited by TSA. Birthdating experiments in the mouse (34) have shown that the vast majority of bipolar cells are generated postnatally from mitotic progenitor cells. The observation of additional bipolar cells following TSA treatment suggests that the bipolar cells in the TSA-treated cultures resulted from fate effects on cells that were postmitotic when the cultures were initiated, and were most likely those cells normally fated to be rods.

Although the function of the transcription factors controlling rod development has been revealed, little is known regarding the regulation of their expression. The results of this study demonstrate that HDACs play an important role in regulating their expression at the initial stages of rod development. Clearly, HDAC activity is required for the expression of a panel of pro-rod genes. The maintenance of expression of Nrl and Crx in the mature retina also requires HDACs, indicating that the function of HDACs in controlling retinal gene expression extends beyond the early developmental stage.

HDACs are generally described as transcriptional repressors. Class I HDACs are ubiquitously expressed in the nucleus. Class II HDACs are capable of shuttling in and out of the nucleus in response to signals. The association of Class II HDACs with the transcription factor MEF2 repressed cardiomyocyte differentiation through the silencing of MEF2-mediated gene transcription. Phosphorylation of Class II HDACs (HDAC 4, 5, 7) caused them to move from the nucleus to the cytoplasm, thus releasing transcription repression, and allowing myocytes to differentiate. However, recent studies have implicated HDACs in the process of transcription activation, providing evidence that HDACs can also act as transcription activators, inducing interferon-stimulated gene expression and the subsequent antiviral response [21-24]. These new findings strongly suggest that the relative acetylation level of HDAC target proteins, regulated by the opposing acitivity of HDACs and HATs, determines the transcription states of specific genes, either activating or repressing transcription. Our results support a role of HDACs as transcription activators, as they may directly regulate the promoter activity of Nrl and Crx. However, from the reporter analysis of Nrl and Crx promoters, we can not completely exclude the possibility that TSA acted indirectly on the Nrl and Crx promoters via activation of transcription repressors that in turn act on the Nrl and Crx promoters. In the latter case, a transcription repressor protein would need to be induced, whose expression is normally repressed by HDACs. Given the fast action of TSA to nearly abolish the expression of Nrl and Crx within 3 hours, even in the presence of a protein synthesis inhibitor cycloheximide, it is more likely that the HDACs are transcription activators of these key retinal development regulators.

It is known that HDACs have many non-histone target proteins. Some are nuclear transcription factors, such as GATA4, NFAT, RB, and p53; others are cytoplasmic targets, such as tubulin and hsp90, which were identified as substrates for HDAC6. HDAC6 deacetylates α-tubulin, with a resulting increase in cell motility [11]. Recently, it was found that HDAC6 mediated acetylation controls the chaperone function of heat shock protein 90 [12,14]. The biological function of other Class II HDACs, when they are in the cytoplasm, remains unknown. In our study, TSA treatment resulted in an increased acetylation of histones and other cellular proteins, including tubulin and two other species that are unknown. Interestingly, although TSA-induced histone acetylation was barely appreciable after 3 hours, a significantly enhanced acetylation of non-histone targets was obvious in 3 hours, and the acetylation levels of these non-histone targets was maintained at later time points. The fast acetylation of non-histone targets is consistent with the kinetics of the mRNA downregulation by TSA. These results suggest that modification of non-histone targets by HDACs may mediate an important pathway in the regulation of retinal gene expression. Using a high-affinity antibody against acetyl-lysine groups will facilitate the purification and identification of these non-histone targets of HDACs by mass spectrometry.

Given the multiplicity of family members from the Class I and Class II HDACs, it is desirable to identify individual HDACs responsible for their role in gene expression regulation. With the development of inhibitors specific to individual HDAC family members [45], it should be possible to address this question in the retina, as well as in other tissues. In addition, analysis of HDAC knockout mice will aid in the identification of the functions of individual HDACs.

## Conclusion

Inhibition of HDACs by the drug, TSA, led to a dramatic reduction in the differentiation of the most abundant retinal cell type, the rod photoreceptor. Rod differentiation was completely absent when HDAC activity was blocked. Key regulators of rod development, the transcription factors Crx, Nrl, Otx2, and others, were greatly reduced within 3 hours of the application of the drug. This appeared to be at least in part due to repression of transcription, as we luciferase assays with the Crx and Nrl regulatory regions showed that transcription from these regions was negatively regulated by TSA. No new protein synthesis was required, suggesting that HDAC activity might directly positively regulate these promoters. The cellular outcome of TSA application may indicate a cell fate switch in that rod photoreceptors and Muller glia were completely absent from treated cultures, while an interneuronal cell type, the bipolar cell, was significantly increased. In addition, cell death was increased, and cell proliferation was completely blocked by TSA.

## Methods

### RT-PCR analysis of retinal HDAC expression

Random-primed reverse transcription was carried out with 2 μg of total RNA isolated from P2 mouse retinal explants using Superscript II (Invitrogen). The resultant cDNA was used in a 30-cycle PCR amplification for HDAC1 with forward primer (5'-AGCAAGATGGCGCAGACTCAG-3') and reverse primer (5'-GGCCAACTTGACCTCTTCTTTG-3'), HDAC3 with forward primer (5'-ATGGCCAAGACCGTGGCGTAT-3') and reverse primer (5'-ACTTTCCTTGTCGTTGTCATG-3'), HDAC4 with forward primer (5'-AAGAAGCTTGTGGCAACTTG-3') and reverse primer (5'-TGAGTTGAGTGGTTTACACG-3'), HDAC5 with forward primer (5'-CAAACACTGGAGCTGTGTAC-3') and reverse primer (5'-TCCATGGGCTCCTCTGCTGG-3'), and HDAC6 with forward primer (5'-TTTCCCTTCTGAGGCCACAG-3') and reverse primer (5'-TCCTTCTGGGTAGAACAGAG-3'). PCR products were cloned into PCR2.1 vector (Invitrogen), and sequenced using M13 forward primer.

### Northern blot analysis

Approximately 10 μg of total RNA was isolated with Trizol (Invitrogen) from retinal explant cultures treated with either 1 μM TSA (Upstate Cell Signaling Solutions), or 20 μg/ml cycloheximide (Sigma), or DMSO for vehicle-treatment control. Northern blot analysis was performed as previously described [42].

### Microarray analysis

Retinal explants were cultured on filters as previously described [46]. Cultured explants were treated with either 1 μM TSA or DMSO for 3 hours. Total RNA was extracted with Trizol (Invitrogen). Approximately 10 μg of total RNA was used in reverse transcription reaction (Superscript II from Invitrogen) to label cDNA by incorporating Cy3- or Cy5-dCTP (Amersham Pharmacia). Cy3- and Cy5- labeled probes were combined together and hybridized to a cDNA microarray, consisting of 11,136 clones from the Brain Molecular Anatomy Project clone set provided by Dr. Bento Soares (University of Iowa) and over 600 retinal cDNA clones collected in our laboratory [47] and printed by Biogen (Service kindly provided by Jeff Shearstone and Steve Perrin). After an overnight hybridization at 42°C, microarray chips were washed in 0.2XSSC/0.1%SDS at room temperature, followed by two room temperature washes in 0.2XSSC. Hybridized chips were scanned with an Axon GenePix scanner (Axon Instruments) and acquired images were analyzed using the GenePix software package (Axon Instruments).

### Luciferase reporter assay by in vitro electroporation

7 kb Nrl (NCBI: NT_039606, 28897897–28904977, Matsuda and Cepko, [48]) and 5 kb Crx (NCBI: NT_109951, 13369–18369, Matsuda and Cepko, [48]) promoter sequences were cloned into firefly luciferase plasmid pGL3-basic (Promega). Promoter luciferase reporter constructs were co-transfected with pCAG-renilla luciferase plasmid into P2 mouse retinae using an in vitro electroporation technique [49]. In short, 1.0 μg/μl of promoter firefly luciferase and 0.5 μg/μl pCAG-renilla luciferase plasmids were prepared in PBS and placed into a microelectroporation chamber (Nepagene, model CUY532) that contained dissected mouse retinae. Unidirectional electric pulses were applied at 50 milliseconds 5 times, each of which was followed by a 950 milliseconds pause. Electroporated retinae were recovered in an overnight culture before being treated with either 1 μM TSA or DMSO for 24 hours. Retinal lysates were prepared and used for a dual luciferase assay following the instructions from the manufacturer (Promega).

### Western blot analysis

P2 mouse retinal explants were cultured in the presence of 1 μM TSA for 3 and 24 hours. Protein lysates were extracted with 1% TritonX-100 TBS containing 20 mM Tris-Cl, pH 7.4, 150 mM N_a_Cl, 1 mM EDTA, and cocktail protease inhibitor mix (Roche). Extracted proteins were resolved on 4–20% Tris-glycine SDS PAGE and transferred to nitrocellulose membrane followed by immunoblot with antibodies against actyl-lysine (Cell Signaling Technology), actyl-tubulin (Sigma), and α-tubulin (Sigma). Bound primary antibodies were visualized by HRP-conjugated secondary antibody and Supersignal Detection Methods (Pierce).

### TUNEL assay

P2 mouse retinal explants were treated with 100 nM TSA or DMSO for 20 hours, fixed with 4% paraformaldehyde in PBS, pH 7.4, and equilibrated in 30% sucrose in PBS. Cryosections were permeabilized with 0.1% Triton X-100 in 0.1% sodium citrate for 2 minutes on ice before incubation with TUNEL reaction mixture (Roche) for 1 hour at 37°C. DAPI counterstained sections were washed 3 times with PBS and analyzed by fluorescence microscopy.

### BrdU labeling and detection

P2 mouse retinal explants were treated with 100 nM TSA or DMSO for 20 hours, and then were labeled by 10 μM BrdU (Roche) in culture medium for 1 hour at 37°C. Retinal explants were fixed in 4% paraformaldehyde and equilibrated in 30% sucrose in PBS. Cryosections were treated with 4N HCl for 10 minutes and washed three times with PBS. BrdU positive cells were detected by anti-BrdU immunostaining (Roche) and fluorescence microscopy.

### Dissociated cell immunofluorescence

P2 mouse retinal explants were cultured in the presence of either 100 nM TSA or DMSO. After 8 days in culture, retinal explants were collected and washed in PBS. Cleaned explants were dissociated with Papain (Worthington Biochemical Corporation) according to manufacturer's instructions. Dissociated cells were fixed onto glass slides and stained with antibodies against: Rhodopsin, Rho4D2 (mouse monoclonal from Dr. R. Molday [50], 1:200), Chx10 (rabbit polyclonal from our lab, 1:500), PKCα (mouse monoclonal from Oncogene, 1:100), Pax6 (mouse monoclonal from University of Iowa Developmental Studies Hybridoma Bank, 1:500), GS (mouse monoclonal from Chemicon, 1:500), CyclinD3 (rabbit polyclonal from Santa Cruz, 1:200), and ApoE (goat polyclonal from Santa Cruz, 1:100). After several washes with PBS, positively-stained cells were identified by corresponding Cy3-conjugated secondary antibodies (Jackson Immunoresearch Laboratory). Cells were counterstained with DAPI and visualized with Nikon Eclipse E1000 microscope.

## Authors' contributions

BC participated in the design of the study, performed the experimental procedures, and wrote the manuscript. CLC conceived of the study, and participated in its design and coordination and helped to draft the manuscript. All authors read and approved the final manuscript.
